# Classification of multi-differentiated liver cancer pathological images based on deep learning attention mechanism

**DOI:** 10.1186/s12911-022-01919-1

**Published:** 2022-07-04

**Authors:** Chen Chen, Cheng Chen, Mingrui Ma, Xiaojian Ma, Xiaoyi Lv, Xiaogang Dong, Ziwei Yan, Min Zhu, Jiajia Chen

**Affiliations:** 1 College of Information Science and Engineering, Xinjiang University, Northwest Road, Shayibake District, Urumqi, 830046 Xinjiang China; 2 College of Software, Xinjiang University, Urumqi, 830046 China; 3 Key Laboratory of Signal Detection and Processing, Xinjiang University, Urumqi, 830046 China; 4 Xinjiang Medical University Cancer Hospital, Suzhou East Road, Xinshi District, Urumqi, 830011 Xinjiang China; 5 Department of Pathology, Karamay Central Hospital of XinJiang Karamay, Karamay, 834000 Xinjiang Uygur Autonomous Region China; 6 Changji Vocational and Technical College, Urumqi, 830011 China

**Keywords:** Histopathological images of liver cancer, SENet, Degree of differentiation of the whole type, Intelligent classification

## Abstract

**Purpose:**

Liver cancer is one of the most common malignant tumors in the world, ranking fifth in malignant tumors. The degree of differentiation can reflect the degree of malignancy. The degree of malignancy of liver cancer can be divided into three types: poorly differentiated, moderately differentiated, and well differentiated. Diagnosis and treatment of different levels of differentiation are crucial to the survival rate and survival time of patients. As the gold standard for liver cancer diagnosis, histopathological images can accurately distinguish liver cancers of different levels of differentiation. Therefore, the study of intelligent classification of histopathological images is of great significance to patients with liver cancer. At present, the classification of histopathological images of liver cancer with different degrees of differentiation has disadvantages such as time-consuming, labor-intensive, and large manual investment. In this context, the importance of intelligent classification of histopathological images is obvious.

**Methods:**

Based on the development of a complete data acquisition scheme, this paper applies the SENet deep learning model to the intelligent classification of all types of differentiated liver cancer histopathological images for the first time, and compares it with the four deep learning models of VGG16, ResNet50, ResNet_CBAM, and SKNet. The evaluation indexes adopted in this paper include confusion matrix, Precision, recall, F1 Score, etc. These evaluation indexes can be used to evaluate the model in a very comprehensive and accurate way.

**Results:**

Five different deep learning classification models are applied to collect the data set and evaluate model. The experimental results show that the SENet model has achieved the best classification effect with an accuracy of 95.27%. The model also has good reliability and generalization ability. The experiment proves that the SENet deep learning model has a good application prospect in the intelligent classification of histopathological images.

**Conclusions:**

This study also proves that deep learning has great application value in solving the time-consuming and laborious problems existing in traditional manual film reading, and it has certain practical significance for the intelligent classification research of other cancer histopathological images.

## Background

Liver cancer is one of the malignant tumors with extremely high fatality rate and directly threatening human life. Hepatocellularcarcinoma (HCC) is a kind of primary liver cancer derived from hepatocytes, with a high degree of malignancy. There are approximately 750,000 patients worldwide each year. The mortality rate of HCC is as high as 93%, ranking third among all malignant tumors. In 2012, more than 782,500 new liver cancer cases were diagnosed, and more than 745,000 liver cancer-related deaths were recorded globally; of these, half of the total numbers of cases and deaths occurred in China [1–8]. In recent years, the global morbidity and mortality of liver cancer have continued to increase, and liver cancer has seriously threatened human life and health. The main clinical features of liver cancer are low predictability, rapid deterioration, and easier death after cancer. The key to the treatment of liver cancer lies in timely diagnosis, and the diagnosis of liver cancer is very important [9–13]. At present, the main diagnostic methods for liver cancer include serum test, biopsy, and imaging diagnosis. Among them, the histopathological images of liver cancer can clearly see the location, size, number, cell and histological type, degree of differentiation, vascular and capsular invasion, satellite foci and metastases, and the lesions of the liver tissue adjacent to the cancer. Among them, the degree of differentiation can reflect the degree of malignancy. The higher the degree of differentiation, the closer to the normal tissue cells, the lower the degree of malignancy. The lower the degree of differentiation, the higher the degree of malignancy. The diagnosis and treatment of different degrees of differentiation is crucial to the survival rate and survival time of patients [14, 15]. Therefore, the accurate classification of histopathological images of liver cancer has a decisive and irreplaceable role in the diagnosis of liver cancer with different degrees of differentiation. However, the classification of histopathological images with different degrees of differentiation has problems such as time-consuming, labor-intensive, and large manual investment. At the same time, due to the lack of experience of doctors or the fatigue caused by the doctors working for a long time and the individual's subjective consciousness are likely to cause misjudgment, which seriously affects the formulation of the patient's treatment plan and prognostic effect [16, 17]. It has important research value for the further study of histopathological image classification of liver cancer [18–20].

In recent years, with the rapid development of artificial intelligence, artificial intelligence algorithms have been widely used in the medical field. Deep learning, as one of artificial intelligence algorithms, is a machine learning method based on deep neural networks [21, 22]. Deep learning is widely used in computer vision and natural language processing tasks [23]. Especially in medical image processing and early disease diagnosis [24–34]. Krishan et al. used six different classifiers to classify different stages of tumors from CT images. The accuracy of tumor classification ranged from 76.38 to 87.01%. This will allow radiologists to further save valuable time before liver diagnosis and treatment [35]. However, this study still has shortcomings such as the need to improve the classification accuracy.YU-SHIANG LIN used a GoogLeNet (Inception-V1)-based binary classifier to classify HCC histopathology images. The classifier achieved 91.37% (± 2.49) accuracy, 92.16% (± 4.93) sensitivity, and 90.57% (± 2.54) specificity in HCC classification [36]. In the work of Yasmeen Al-Saeed, a computer-aided diagnosis system was introduced to extract liver tumors from computed tomography scans and classify them as malignant or benign. The proposed computer aided diagnosis system achieved an average accuracy of 96.75%, sensitivity of 96.38%, specificity of 95.20% and Dice similarity coefficient of 95.13% [37]. However, this study still has shortcomings such as a single sample type. Xu et al. developed a radiomic diagnosis model based on CT image that can quickly distinguish Hepatocellular carcinoma (HCC) from intrahepatic cholangiocarcinoma (ICCA), in the training set, the AUC of the evaluation of the radiomics was 0.855 higher than for radiologists at 0.689. In the valuation cohorts, the AUC of the evaluation was 0.847 and the validation was 0.659, which may facilitate the differential diagnosis of HCC and ICCA in the future [38]. However, this study still has shortcomings such as the need to improve the classification accuracy. Wan et al. propose a deep learning-based multi-scale and multi-level fusing approach of CNNs for liver lesion diagnosis on magnetic resonance images, termed as MMF-CNN. They apply proposed approach to various state-of-the-art deep learning architectures. The experimental results demonstrate the effectiveness of their approach [39]. Zhou et al. introduced basic technical knowledge about AI, including traditional machine learning and deep learning algorithms, especially convolutional neural networks, and their use in liver disease medical imaging Clinical applications in the field, such as detecting and evaluating focal liver lesions, promoting treatment and predicting liver response to treatment. Finally, it is concluded that machine-assisted medical services will be a promising solution for liver medical services in the future [40]. Li, Jing designed a fully automated computer-aided diagnosis (CAD) system using a convolutional neural network (CNN) network structure to diagnose hepatocellular carcinoma (HCC). The study used a total of 165 venous phase CT images (including 46 diffuse tumors, 43 nodular tumors and 76 large tumors) to evaluate the performance of the proposed CAD system. Finally, the CNN classifier was obtained for the classification accuracy of diffuse, nodular and massive tumors of 98.4%, 99.7% and 98.7%, respectively [41]. However, the study still has sample types that do not use the gold standard, and it is difficult for doctors to design and arrange accurate treatment procedures based on the diagnosis results. Azer, Samy A studied the role of CNN in analyzing images and as a tool for early detection of HCC or liver masses. The results of this study prove that the accuracy of CNN for liver cancer image segmentation and classification has reached the best level [42]. However, this study still has the disadvantage of too few classification types, which has certain limitations. Liangqun Lu and Bernie J. Daigle, Jr et.al used the pre-trained CNN model VGG 16, Inception V3 and ResNet 50 to accurately distinguish normal samples from cancer samples using image features extracted from HCC histopathological images. However, the study still has the deficiency of a single classification sample category [43]. Sureshkumar et al. outlined various liver tumor detection algorithms and methods for liver tumor analysis. Proposed deep learning methods such as probabilistic neural networks to detect liver tumors and diagnose them through experimental results, and compare them with different methods. This study proposes a framework that can arrange CT images of the liver in expected or strange ways and distinguish unusual tumors with a reduced false positive rate and expanded accuracy [44]. However, the study still has insufficient sample types, which is not a deficiency of the diagnostic gold standard. In the study of Lin, HX, etc., 217 combinations of two-photon excitation fluorescence and second harmonic generation images were used to train a convolutional neural network based on the VGG-16 framework; the classification accuracy of liver cancer differentiation levels exceeded 90%. It is proved that the fusion of multiphoton microscope and deep learning algorithm can classify the differentiation of HCC, thereby providing an innovative computer-aided diagnosis method [45]. The research is relatively new, but there is still room for improvement in accuracy. In this study, we used a very complete dataset of histopathological images of multi-differentiated HCC, added the visual attention mechanism to the deep learning model to extract the feature information of medical images more effectively, and finally obtained 95.27% in SENet accuracy. This study proves that the SENet deep learning model has good application value in the intelligent classification of histopathological images.

In order to ensure the authenticity and integrity of the data, we have developed a complete data acquisition scheme to obtain a higher quality data set for the classification experiment. Since histopathological images have more obvious and different characteristic information than other conventional natural images, the characteristics of histopathological images must be fully considered when establishing a deep learning model for histopathological image classification. Therefore, this paper adds the visual attention mechanism to the deep learning model. The attention mechanism increases the interpretation of the model by visualizing the attention, which can extract the feature information of medical images more effectively. On this basis, for the first time, we applied the SENet deep learning model to the intelligent classification study of all types of differentiated liver cancer histopathological images, and compared it with the four deep learning models of VGG16, ResNet50, ResNet50_CBAM, and SKNet. Finally, SENet was obtained the classification accuracy rate is 95.27%. The experiment has proved that the SENet deep learning model has a good application prospect in the intelligent classification of histopathological images. The effective classification of different levels of differentiation is convenient for doctors to formulate treatment plans more effectively, so that patients can get treatment in time. At the same time, it can effectively reduce the workload of medical workers and reduce the reading time of histopathological images of liver cancer. This also provides a certain reference value for deep learning in the research of intelligent classification of medical images.

## Materials and methods

### Obtaining slice samples

The Institutional Review Committee of the Cancer Hospital Affiliated to Xinjiang Medical University approved this study with the ethical approval number K-2021050. Male and female patients who were treated in the Cancer Hospital of Xinjiang Medical University from August 2020 to August 2021 participated in this study with informed consent. Fresh liver cancer tissue samples were collected by professional surgeons. All patients did not receive hormone therapy, radiotherapy or chemotherapy before surgery. In the end, without collecting the personal information of these patients, we obtained 74 liver cancer tissue samples from these patients (including: 24 poorly differentiated pathological slice samples, 28 moderately differentiated pathological slice samples, and 22 highly differentiated pathological slice samples). The following is the specific process of slice sample preparation:

The first step is fixation. Fresh liver cancer specimens are quickly fixed with 10% neutral buffered formalin at room temperature for 18–24 h. The second step is dehydration, by immersing the formalin-fixed sample in a series of alcohol solutions that increase with the alcohol concentration to remove water from it. After removing the dehydrated sample with an organic solvent, the third step of sample preparation is to bury it in molten paraffin. After cooling, each paraffin-embedded block (1.5 × 1.5 × 0.3 cm^3^) was sliced into 4 µm consecutive slices, and each slice was placed on an albumin glass slide. The fourth step dyeing starts after the paraffin is dissolved. The histological section of liver cancer tissue is stained with H&E, which is the most commonly used staining method in medical diagnosis. Finally, 3 skilled pathologists with more than 10 years of pathology experience examined histological sections under a light microscope and selected representative H&E sections. The diagnosis results were consistent. Table 1 shows the basic information of all patients.

**Table 1:** Basic patient information

Differentiation	Age	Gender		Staging				Alcoholism		Smoking	
		M	W	I	II	III	IV	Y	N	Y	N
Poorly differentiated	35–50	7	3	4	1	3	2	2	8	5	5
	51–66	6	1	4	1	1	1	2	5	3	4
	67–82	4	3	2	4	1	0	0	7	0	7
Moderate differentiation	35–50	8	0	2	3	3	0	2	6	3	5
	51–66	12	2	8	4	2	0	6	8	8	6
	67–82	5	1	1	0	5	0	2	4	3	3
Well differentiated	35–50	2	0	1	0	0	1	2	0	2	0
	51–66	12	1	11	2	0	0	1	12	5	8
	67–82	4	1	1	1	3	0	0	5	2	3

### Data set preparation

**Fig. 1:**
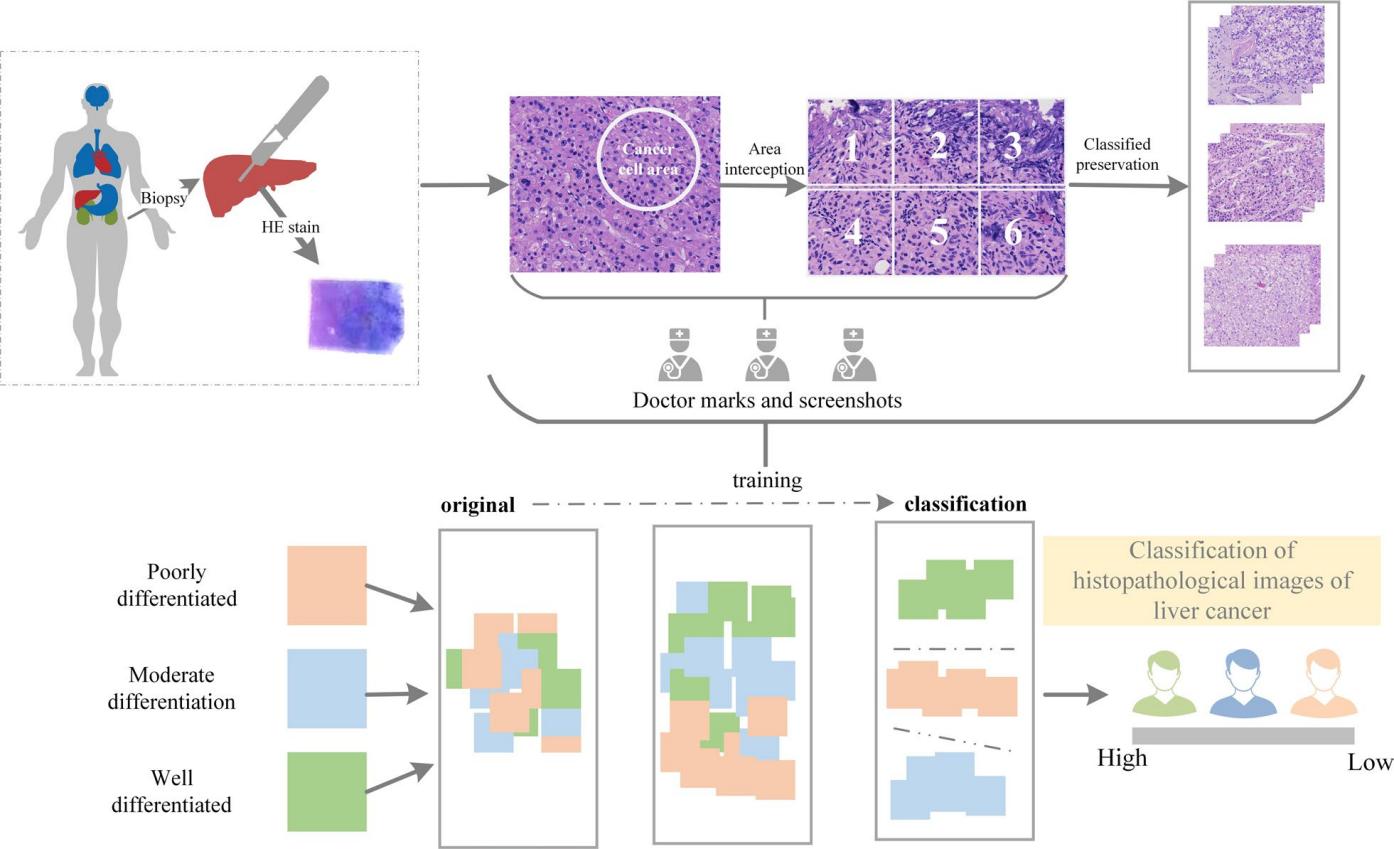
Flowchart of data acquisition

In this paper, 444 liver cancer histopathological images (There are 144 poorly differentiated histopathological images, 168 moderately differentiated histopathological images, and 132 highly differentiated histopathological images.) were obtained to form the research data set, and every image acquisition was done in the digital pathology scanner (Model: PRECICE 500B, Shanghai, China) with size of 1665 × 1393 pixels. The specific flow chart as is shown in Fig. 1.

The data set preparation process is as follows:

1. Putting 5 liver cancer pathological slices with the same degree of differentiation into the card slot of the digital pathology scanner simultaneously to complete the high-definition scanning of the pathological slices.
2. The dense area of Cancer cell in the high-definition image should be marked based on pathological expertise by a professional pathologist and cutting off 6 images evenly in a 2 × 3 matrix order in it after scanning, and then save the current images.
3. Repeat step (2) for the 5 pathological slices in the card slot in turn, and repeat the above process until all images are acquired.
4. The pathology medical workers should verify again whether all the acquired images meet the classification of different degrees of differentiation, exclude all images without normal cells, and perform the next classification experiment after confirming that they are correct.

### Experimental environment

All program codes in the study, are developed based on the Python language. The software and hardware conditions of the specific experimental environment are shown in Table 2.

**Table 2:** Experimental environment

Category	Name	Version
CPU	IntelCore I5 9600KF	–
RAM	32G 2666 MHz	–
GPU	NVIDIA GTX 1070	–
Development language	Python	V3.7.0
Image processing library	Sci-kit image	V0.16.2
Data processing library	Numpy	V1.17.2
Deep learning framework 1	Sci-kit learn	V0.22
Machine learning library	TensorFlow	V2.1.0
Deep learning framework 2	Keras	V2.3.1

### Experimental methods

#### Image enhancement

There are 444 original liver cancer histopathological images used in this paper, which are divided into three categories: poorly differentiated, moderately differentiated, and well differentiated. Because a large number of samples are needed for training to achieve better generalization in the subsequent deep learning model training. Obviously, due to raw data set used in this paper can not meeting training conditions, so we have to enhance the raw data set. However, each piece of the histopathological image of liver cancer contains rich pathological diagnosis information, and many image enhancement methods cannot well retain its pathological diagnosis information. At the same time, in order to restore the complete process of medical workers in actual reading as much as possible, this research adopts image rotation enhancement method to enhance the original image, the principle is as follows:

Image rotation: Suppose that $\left({x}_{0},{y}_{0}\right)$ is the coordinate after rotation, $\left(x,y\right)$ is the coordinate before rotation. $\left(m,n\right)$ is the center of rotation. $\theta$ is the angle of rotation. $\left(left,top\right)$ is the coordinates of the upper left corner of the rotated image. The realization formula is as shown in (1):

1 $$\left[ {\begin{array}{*{20}c} {x_{0} } & {y_{0} } & 1 \\ \end{array} } \right] = \left[ {\begin{array}{*{20}c} x & y & 1 \\ \end{array} } \right]\left[ {\begin{array}{*{20}c} 1 & 0 & 0 \\ 0 & { - 1} & 0 \\ { - m} & n & 1 \\ \end{array} } \right]\left[ {\begin{array}{*{20}c} {\cos \theta } & { - \sin \theta } & 0 \\ {\sin \theta } & {\cos \theta } & 0 \\ 0 & 0 & 1 \\ \end{array} } \right]\left[ {\begin{array}{*{20}c} 1 & 0 & 0 \\ 0 & { - 1} & 0 \\ {left} & {top} & 1 \\ \end{array} } \right]$$

The rotation angle in this paper is set to 10°, and each original image is rotated at an angle of 10° each time. A picture is rotated 36 times, and a total of 36 images are obtained after each original image is rotated. The enhanced data set contains a total of 15,984 histopathological images.

#### Model training

**Fig. 2:**
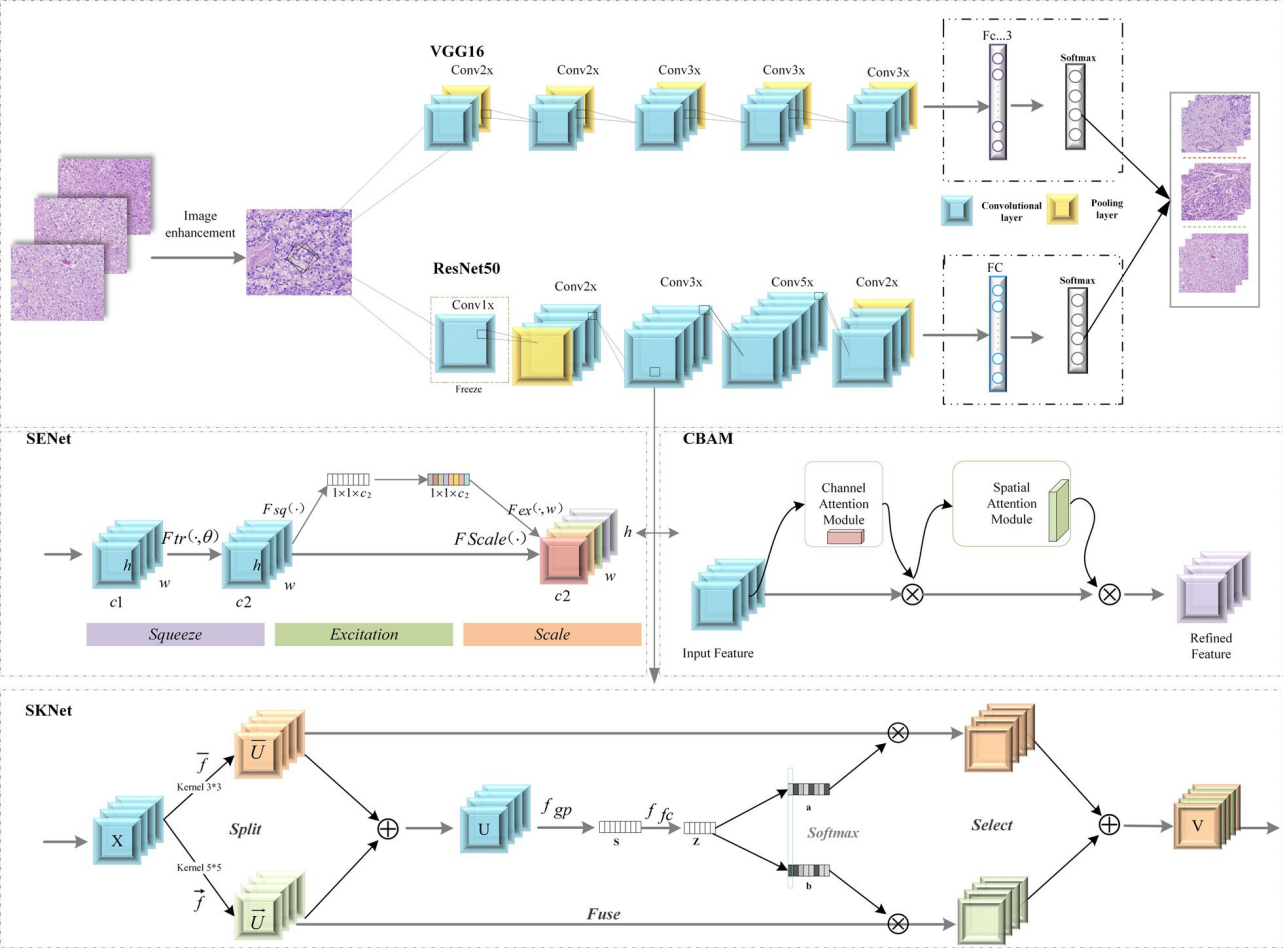
Experimental frame diagram

In this paper, five deep learning models which are VGG16, ResNet50, ResNet_CBAM, SENet, and SKNet are selected for classification experiments [46, 47]. The above model and specific training process will be described in detail as follows, and the experimental frame diagram is shown in Fig. 2.

VGG16 network is relatively simple and does not have so many hyperparameters. It is a simple network that only needs to focus on building a convolutional layer. First, a 3 × 3 filter with a stride of 1 is used to construct a convolutional layer, and the padding parameter is the parameter in the same convolution. Then use a 2 × 2 filter with a stride of 2 to build the maximum pooling layer. Therefore, a major advantage of VGG network is that it does simplify the neural network structure. However, the amount of parameters is too large, which consumes a lot of computing resources, so it is not suitable for image learning with complex features [48, 49].

Traditional convolutional networks or fully connected networks will more or less have problems such as information loss and loss when information is transmitted, and at the same time, they can cause gradients to disappear or explode, making deep networks unable to train. ResNet solves this problem to a certain extent. Its main idea is to add the idea of Highway Network to the network, which allows to retain a certain proportion of the output in the previous network layer. The input information is directly detoured to the output to protect the integrity of information in ResNet. The entire network only needs to learn the part of the difference between input and output to simplify the learning objectives and difficulty [50, 51]. Based on the above advantages, this article chooses ResNet50 as one of the training models.

CBAM is a simple and effective convolutional neural network attention module. Given an intermediate feature map arbitrarily in the convolutional neural network, CBAM injects the Attention Mapping along two independent dimensions of the channel and space of the feature map, and then multiplies the Attention by the input feature map to perform Adaptive feature refinement on the input feature map. CBAM is an end-to-end universal module [52]. Based on the advantages of CBAM combined with the ResNet50 model, this paper selects the ResNet_CBAM model for classification experiments.

The idea of SENet is very simple, and it is easy to extend to the existing network structure. The SENet model proposes a new block structure, called the SE block, which uses each feature layer in the squeeze compression model structure, and uses excitation to capture feature channel dependence [53, 54].So the SE block can be combined with many existing models. The SE module mainly includes two operations, Squeeze and Excitation, which can be applied to any mapping $F_{tr} :X\, \to \,U$, $X \in R^{{H^{\prime } \times Y^{\prime } \times M^{\prime } }}$, $U\, \in \,R^{H \times Y \times M}$ take convolution as an example, the kernel of convolution is $V = \left[ {v_{1} ,v_{2} , \ldots v_{m} } \right]$, where v_m represents the *m*th convolution kernel. Then output $U = \left[ {u_{1} ,u_{2} , \ldots u_{M} } \right]$;

2 $$u_{m} = v_{m} {*}X = \mathop \sum \nolimits_{n = 1}^{{M^{\prime } }} v_{m}^{n} {*}x^{n}$$

* represents the convolution operation, and $v_{m}^{n}$ represents an n channel 2-D convolution kernel, which inputs the spatial characteristics of a channel and learns the feature space relationship, but because the convolution results of each channel are added, the feature relationship of the channel is mixed with the spatial relationship learned by the convolution kernel. The SE block is to remove this confounding, so that the model directly learns the feature relationship of the channel.

SKNet and SENet are lightweight modules that can be directly embedded in the network. Feature maps of different convolution kernelsizes are fused through the Attention Mechanism, and the size of the Attention is based on the deterministic information extracted by convolution kernels of different sizes [55]. Intuitively, SKNet assimilate a soft attention mechanism into the network, so that the network can obtain information of different Receptive Field, which may become a network structure with better generalization ability [56].

After selecting the experimental model, this article randomly divides the ratio of the training set and the test machine according to the ratio of 8:2, and the specific division is shown in Table 3.

**Table 3:** Data partition

Differentiation type	Raw data	Enhanced data	Training set	Test set
Poorly differentiated	144	5184	4176	1008
Moderate differentiation	168	6048	4860	1188
Well differentiated	132	4752	3816	936

In this paper, all the deep model training batches are set to 300 Epoch, and the configuration of the optimizer and the setting of the learning rate are also crucial in model training. Based on the advantages of the (Stochastic gradient descent) SGD method in the application of large data sets, the training speed is very fast and as long as the noise is not very large, it can converge well. This paper chooses the SGD method as the optimizer configuration algorithm. The algorithm principle is as follows:

Suppose there are n samples in a batch, and randomly select one ${i}_{s}$. The model parameter is set to W, the cost function is set to $\mathrm{J}\left(\mathrm{W}\right)$, the gradient is $\Delta \mathrm{J}\left(\mathrm{W}\right)$, and the learning rate is ${\upvarepsilon }_{\mathrm{t}}$, then the method update parameter expression is:

3 $$W_{t + 1} = W_{t} - \varepsilon_{t} g_{t}$$

Among them; ${g}_{t}=\Delta {J}_{{i}_{s}}\left({W}_{t};{X}^{\left({i}_{s}\right)};{X}^{\left({i}_{s}\right)}\right),{i}_{s}\in \mathrm{1,2}\cdots n$ represents a gradient direction randomly selected, and ${W}_{t}$ represents the model parameter at the moment, $E\left({g}_{t}\right)=\Delta J\left({W}_{t}\right)$, the expectation is the correct gradient descent.

In the above formula (6), the learning rate ε_t is one of the most important parameters in training, and the learning rate is set to 0.001 in the training of this article.

### Evaluation index

There are many commonly used evaluation criteria for medical image classification, such as accuracy, error rate, false positive rate, true negative rate, false negative rate, true negative rate, etc. The evaluation indexes adopted in this paper include confusion matrix, Precision, recall, F1 Score**,** etc. These evaluation indexes can be used to evaluate the model in a very comprehensive and accurate way. The detailed description is given below.

**1. Confusion matrix**: All evaluation indexes are directly or indirectly related to the confusion matrix. All evaluation indexes can be directly calculated from the confusion matrix, and their specific forms are shown in Table 4:

**Table 4:** Confusion matrix

	Actual class	
	Positive class	Negative class
*Predicted class*		
Positive class	True Positive (TP)	False Positive (FP)
Negative class	False negative (FN)	True negative (TN)

In the confusion matrix shown in the table above:

- TP represents that the actual class is positive, and the predicted class of the model is also positive.
- FP represents that the predicted class is positive, but the actual class is negative. The actual class is inconsistent with the predicted class.
- FN represents that the predicted class is negative, but the actual class is positive. The actual class is inconsistent with the predicted class.
- TN represents that the actual class is negative, and the predicted class of the model is also Negative.

By using the confusion matrix, we can intuitively see the classification of a model in positive and negative categories.

Precision and recall: Formula (4) and formula (5) are used to calculate the precision and recall of the positive class:

4 $$precision = \frac{TP}{{TP + FP}}$$

5 $$recall = \frac{TP}{{TP + FP}}$$

Precision represents the proportion of samples whose actual class is positive in the samples whose predicted class is positive; the recall represents the percentage of samples that are successfully predicted in a truly positive sample by the model.

F1 Score: F1 Score is calculated by Formula (6):

6 $${\text{F1}}\,{\text{Score}} = \frac{2\, \cdot \,precision\, \cdot \,recall}{{precision + recall}}$$

F1 Score takes both the precision and recall of the classification model into account. It can be regarded as a harmonic average of precision and recall of model. It has a maximum value of 1 and a minimum value of 0.

## Results analysis and discussion

### The classification results

In this section, the classification results of different models are analyzed in detail. The training accuracy curves of all classification models under the same training conditions including optimizer algorithm, loss function, learning rate, Epoch and other conditions are shown in Fig. 4. Table 5 shows the classification accuracy of different differentiation types.

**Table 5:** Classification accuracy of histopathological images of liver cancer

Classifier	Poorly differentiated	Moderate differentiation	Well differentiated	Accuracy
VGG16	77.98	79.12	73.08	76.95
ResNet50	96.43	90.99	**95.94**	94.22
ResNet50_CBAM	95.24	**94.28**	94.76	94.73
SENet	**98.41**	92.59	95.30	**95.27**
SKNet	96.43	93.94	95.30	95.15

Bold indicates the highest accuracy

Unit:%

**Fig. 3:**
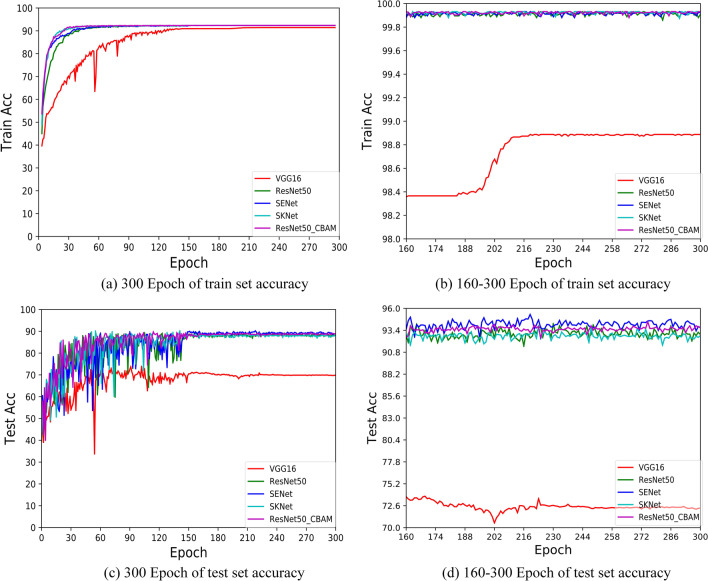
Classification accuracy curves

In Fig. 3a and c, we respectively show the accuracy curves of training set and test set in the five classification models. In order to observe the convergence process in the training process more intuitively, we intercept respectively the accuracy curves of training set and test set in 160–300 Epoch, as shown in Fig. 3b and d. In the training set, the models except VGG16 began to converge when the Epoch was 50 and completely converged when the Epoch was 120, while VGG16 model tended to converge completely when the Epoch was 215. In the test set, the models began to converge at 150 Epoch, and the VGG16 model began to converge at 220 Epoch. This also proves that the simple network model focusing on the construction of convolutional layer is not suitable for the classification task of histopathological images of liver cancer.

Through horizontal comparison of the accuracy of different models in Table 5, it was found that SENet model achieved the best classification effect with a classification accuracy of 95.27%. The ResNet50 model and resnet50_CPAM model achieved respectively 95.94% accuracy in the classification of well differentiated histopathological images and 94.28% accuracy in those of moderate differentiation histopathological images. It also confirmed the superiority of ResNet50 model in the classification of images with relatively simple features. At the same time, the SENet model achieved a 98.41% classification accuracy in the classification of poorly differentiated histopathological images.

### Model evaluation

**Fig. 4:**
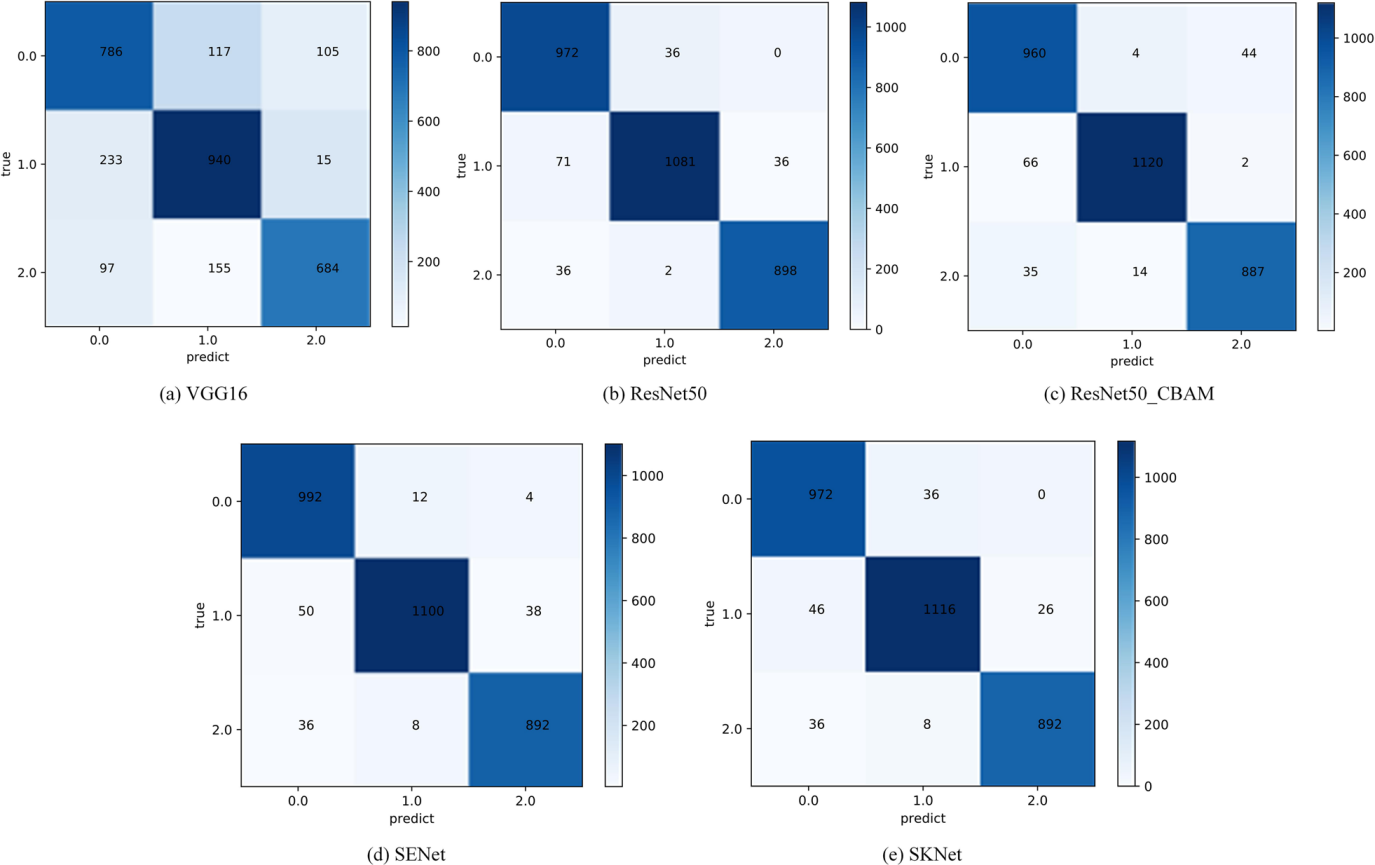
Confusion matrix of different models

In this paper, the confusion matrix of the training model is used as the evaluation index of the model. From the confusion matrix of the model (0 represents poorly differentiated, 1 represents moderate differentiation, 2 represents well differentiated) we can very clearly see the liver cancer histopathological image classification accuracy of different models in different differentiation degree. From the confusion matrix of different models, it can be found that the VGG16 model has a general classification effect on the images with three different degrees of differentiation. It is worth noting that, in the moderate differentiation image classification, the number of images which are misjudged as poor differentiation was much larger than the number of images which are misjudged as well differentiation. The main cause of this phenomenon lies in the relatively complex image characteristics of histopathology images. So the VGG16 model whose structure is relatively simple can not learn more from the image characteristics and it causes the classification effect is poor. Since ResNet50 and ResNet50_CBAM models have deeper structures, their image classification effect with different degrees of differentiation is better than VGG16 model.ResNet50_CBAM model is slightly better than ResNet50 model in the classification of moderately differentiated images, and the number of correct classification of poorly differentiated images and well differentiated images are relatively consistent. SENet and SKNet models have achieved good classification results in the classification of three types of images, and they are completely consistent in the classification of well differentiated images. SENet model has more correct judgments in the classification of poorly differentiated images than SKNet model, while SKNet model has a slight advantage in the classification of moderate differentiation images. The main reason for this phenomenon is that SENet model pays more attention to strengthening useful information and compressing useless information, so it is more suitable for histopathological images with complex features (Fig. 4).

Through further analysis and calculation of the confusion matrix of different models, we can use more representative Precision, Recall and F1 Score to prove the reliability and generalization ability of different models. These indexes can comprehensively describe the performance of the model from different perspectives. Table 6 shows the evaluation indexes of different models in detail.

**Table 6:** Evaluation indexes of different models

Differentiation type	Metrics	VGG16	ResNet50	ResNet50_CBAM	SENet	SKNet
Poorly differentiated	Precision	70.43	90.08	90.48	92.02	**92.22**
	Recall	77.98	96.43	95.24	**99.41**	96.43
	F1 Score	74.01	93.15	92.80	**95.11**	94.28
Moderate differentiation	Precision	77.56	96.60	**98.42**	98.21	96.21
	Recall	79.12	90.99	**94.28**	92.59	93.94
	F1 Score	78.33	93.71	**96.30**	95.32	95.06
Well differentiated	Precision	85.07	96.15	95.07	95.50	**97.17**
	Recall	73.08	**95.94**	94.76	95.30	95.30
	F1 Score	78.62	96.04	94.92	95.40	**96.22**

Bold indicates the highest accuracy

Unit:%

Through the horizontal comparison of the above five classification models and their three different evaluation indexes, it was found that the VGG16 model did not achieve ideal results in any classification of the differentiation degree. In the classification of poorly differentiated histopathological images, SENet model achieved good scores both in recall and F1 Score, and its precision was next only to SKNet model. This indicates that SENet model is more suitable for distinguishing poorly differentiated histopathological images with more complex image features. We analyze the main reason for this phenomenon and think this is because the difference in image features of histopathological images is mainly reflected in channel dimension. While SENet model can pay more attention to channel features with the most information and suppress the channels features that are not important. Resnet50_CBAM model achieved the best score in the classification of moderate differentiation histopathological images in all three indexes. This is thanks to two independent sub-modules of CBAM, Channel Attention Module (CAM) and Spartial Attention Module (SAM). It is more suitable for moderate differentiation images that appear to be clearly abnormal from normal cells, but retain traces of some tissue source. The SKNet model achieved the best score in Precision and F1 Score for image classification of well differentiated histopathological images. For well differentiated histopathological images, they contain more normal cells, and their image features are relatively simple and easy to learn. SKNet carries out an attention mechanism for the convolutional nucleus, that is, the network selects the appropriate convolutional nucleus by itself, without too many changes to the overall structure of the model. Therefore, SKNet model is more stable and has better generalization ability in the classification of well differentiated histopathological images.

## Conclusion

In this paper, a complete data acquisition scheme was developed for the intelligent classification of histopathological images with different degrees of differentiation, and histopathological images of liver cancer with full types and degrees of differentiation were obtained. Because histopathological images have more obvious and different feature information than other conventional natural images, the characteristics of histopathological images must be fully considered when establishing the deep learning model of histopathological image classification. Therefore, this article adds visual attention mechanism to some deep learning model, which can increase interpretation forms of the model through attention visualization and can more effectively extract the characteristic information of the medical image. Five different deep learning classification models are applied to collect the data set and evaluate model. The experimental results show that the SENet model has achieved the best classification effect with an accuracy of 95.27%. The model also has good reliability and generalization ability.

However, our work has the following limits: (1) the scale of the dataset used for the experiments was small, so the methods need to be compared in a larger dataset; (2) The data source is single, and there is still a certain distance from clinical application. In the future, we will continue to collect more samples from more different hospitals to further improve the experiment and make up for the shortcomings of the experiment.

This study also proves that deep learning has great application value in solving the time-consuming and laborious problems existing in traditional manual film reading, and it has certain practical significance for the intelligent classification research of other cancer histopathological images.

## Data Availability

The data that support the findings of this study are available from the Cancer Hospital of Xinjiang Medical University but restrictions apply to the availability of these data, which were used under license for the current study. We do not have the right to disclose data and so are not publicly available. Data are however available from the authors upon reasonable request and with permission of the Cancer Hospital of Xinjiang Medical University.
